# Monitoring Hydrogen-Induced Cracking in Tensile Wires of Flexible Pipes by Acoustic Emission Technique

**DOI:** 10.3390/ma19081524

**Published:** 2026-04-10

**Authors:** Kaíque do Rosário Oliveira, Sergio Luis Gonzalez Assias, Merlin Cristina Elaine Bandeira, Davi Ferreira de Oliveira, Hector Guillermo Kotik, Cesar Giron Camerini

**Affiliations:** 1 Laboratory of Non Destructive Testing, Corrosion and Welding (LNDC), Programa de Pós-Graduação em Engenharia Metalúrgica e de Materiais (PEMM), Universidade Federal do Rio de Janeiro, Rio de Janeiro 21941, Brazil; 2 Laboratório de Mecânica da Fratura (LMF), Programa de Pós-Graduação em Engenharia Metalúrgica e de Materiais (PEMM), Universidade Federal do Rio de Janeiro, Rio de Janeiro 21941, Brazil; 3 Laboratório de Instrumentacao Nuclear (LIN), Universidade Federal do Rio de Janeiro, Rio de Janeiro 21941, Brazil

**Keywords:** hydrogen induced cracking, acoustic emission, flexible pipeline amor wire

## Abstract

This study explored the continuous monitoring of hydrogen-induced cracking (HIC) in high-strength steel tension wires used in metal-based flexible pipes, exposed to a H_2_S-saturated aqueous environment, using acoustic emission (AE) techniques. Armor wire samples were subjected to sour conditions under controlled environments for 24 and 96 h. To reinforce and validate the AE findings, a comprehensive characterization was performed, including X-ray microtomography, optical microscopy, and scanning electron microscopy. The experimental results demonstrated that AE techniques effectively monitored the evolution of HIC damage in the armor wire samples, enabling the identification of distinct damage stages and cracking phenomena. These findings confirm that AE can serve as a valuable complementary tool during HIC testing, optimizing test duration and providing insights into the kinetics of the cracking process.

## 1. Introduction

**Figure 1:**
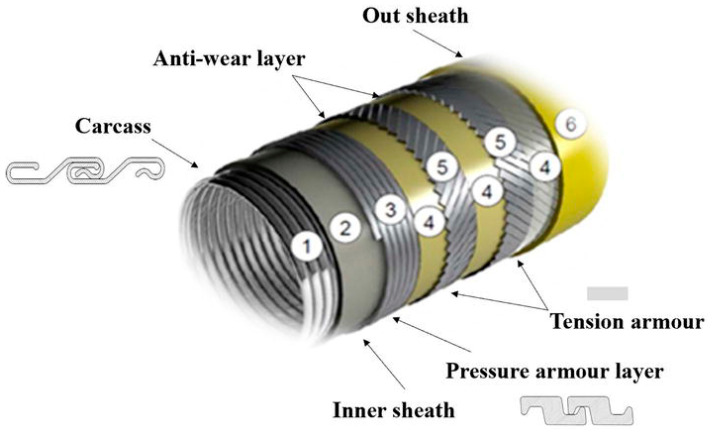
Schematic diagram of the typical structure of a metal-based flexible pipeline. Reproduced under Creative Commons Attribution 3.0 License from [2].

Currently in Brazil, only Petrobras has more than 9000 km of flexible pipelines installed, based on 2023 data [1]. Financial costs and environmental impacts are directly related to failures during the operation of these pipelines, with one of the main causes of damage being corrosive processes in the metallic layers present in the pipeline due to the presence of aggressive gases such as hydrogen sulfide (H_2_S). Figure 1 shows a typical configuration of flexible pipeline, a metal-based flexible pipe. This consists of an internal metallic carcass layer that prevents collapse under external pressure (1), a polymer pressure sheath that provides chemical resistance and fluid containment (2), and one or more armor layers to support mechanical loads, like internal pressure (3) and axial tension (5). Additional layers, including anti-wear tapes (4) and insulation (6), may be incorporated to enhance thermal performance and reduce friction between components.

Hydrogen sulfide can attack the steel wires of the armor layers, promoting hydrogen embrittlement (HE) and stress corrosion cracking (SCC). These mechanisms can cause premature and unexpected failures, compromising the overall mechanical integrity of the pipeline [3,4,5]. Tensile wires are of utmost importance for the metal-based flexible pipeline, as they are responsible for a large part of the tensile strength of the pipes; thus, if this component is damaged, catastrophic failure can occur.

The monitoring of HE and SCC phenomena in these pipelines during operation is particularly challenging and, in some cases, even impossible with the tools currently available. Therefore, the experimental evaluation of hydrogen-induced damage mechanisms under laboratory conditions emerges as a key approach to understanding how these materials would respond under various service conditions.

Laboratory testing offers crucial insights into mechanical behavior and metallurgical characteristics associated with hydrogen-induced cracking (HIC) and SCC in tensile wires of flexible pipelines. These wires operate within a complex annular environment, where the permeation of CO_2_, H_2_S, and water creates a highly corrosive medium that promotes hydrogen-related degradation mechanisms [6]. Recent investigations [7,8] have demonstrated that features, such as high microhardness zones, banded microstructures, and residual stresses from cold drawing, significantly influence the initiation of HIC, even in the absence of non-metallic inclusions.

Further studies suggest that the alignment of cementite and local variations in pearlite morphology may enhance hydrogen trapping, thus increasing the likelihood of crack nucleation [7]. Additionally, Dugstad et al. [6] emphasized that oxygen ingress through the outer sheath, when combined with aqueous H_2_S, results in severe pitting corrosion and SCC under certain annular conditions. Notably, even slight shifts in the annulus environment, including changes in gas composition, operational pressure, or water chemistry, can dramatically alter the dominant degradation mechanisms and accelerate the progression from general corrosion to localized cracking [9].

Standards such as NACE TM0284-2016, “Evaluation of Pipeline and Pressure Vessel Steels for Resistance to Hydrogen-Induced Cracking” [10], provide detailed guidelines and procedures for assessing HIC in steels used for pipelines and pressure vessels.

The standard specifies three saturated H_2_S solutions for testing:

- Solution A: acidified brine solution.
- Solution B: acidified brine.
- Solution C: buffered solution.

For solutions A and B, the test duration is fixed at 96 h. For solution C, the duration depends on the partial pressure of H_2_S but must be at least 96 h. After testing, the standard defines various parameters to evaluate the severity of HIC through analysis of transverse sections of the specimens.

Such laboratory tests are essential for material selection and qualification processes, supporting the development of more robust and reliable flexible pipe systems. However, traditional HIC tests, although standardized and widely applied, are typically long-duration tests, taking 96 h or more (up to 2160 h), and provide only post-mortem information, i.e., damage is only assessed at the end of the test through destructive inspections or non-destructive testing. This limitation hinders the understanding of the timing and evolution of micro-crack initiation and growth [7,8]. In addition, the lack of information during the HIC tests, in many cases, leads to premature interruption of the tests followed by post-mortem evaluation, even when no material degradation has occurred.

Acoustic Emission (AE) has emerged as a promising real-time monitoring technique for laboratory-based corrosion tests. It offers a complementary alternative that may help to build on cracking mechanisms and overcome limitations inherent in conventional evaluation methods for HIC and SCC, since this technique has been proven as effective and powerful in detecting active damage mechanisms during real-time testing [11,12,13,14,15,16,17,18].

**Figure 2:**
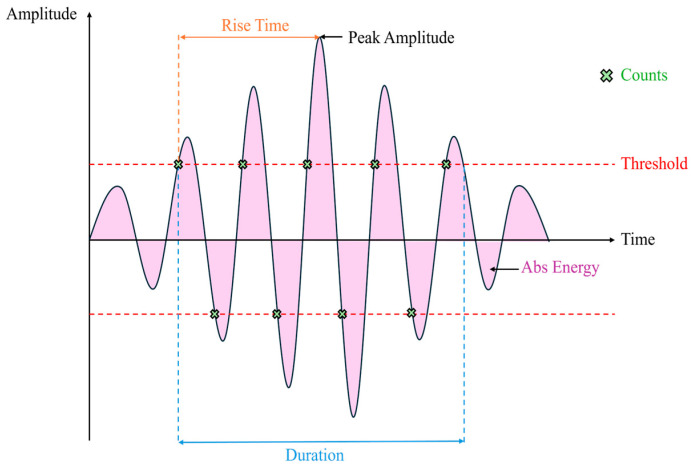
Illustration of acoustic emission parameters.

When a localized event occurs within a material, it generates transient elastic waves that propagate and can be captured by piezoelectric sensors placed on the specimen surface. These AE signals exhibit distinctive features that can be analyzed in both the time and frequency domains, enabling a detailed characterization of the underlying physical phenomena [19,20,21,22,23]. Several key parameters are typically extracted from AE waveforms, as illustrated in Figure 2.

The AE parameters typically serve as the basis for signal classification and damage mechanism identification, providing insight into processes such as hydrogen bubble formation, crack initiation, and propagation [24,25].

From the standpoint of corrosion, HIC, and SCC test, AE sources are typically caused by the evolution of the corrosion and hydrogen embrittlement process, such as bubble production, crack initiation and propagation, and formation of corrosion products, among others [13].

The use of AE in monitoring corrosion tests can be traced back to the 1970s [11,26], in which different materials were immersed in acidic solutions, and the activity of the signals was monitored, establishing their relationships with crack propagation and the volume of hydrogen gas released. Studies to associate the types of phenomena present in corrosion tests with AE signal parameters emerged in the following decades. For example, Pollock et al. [13] indicated the possibility of detecting phenomena such as surface film breakage, gas evolution, microcracks, embrittlement, and crack propagation.

Moreover, different types of corrosion mechanics have also been analyzed to be distinguished primarily using parameter analysis, based on signal amplitude and count, revealing differences between signal populations originating from uniform corrosion, pitting, crevice, and SCC [23,27,28].

Some authors evaluated cumulative counts and energy curves to divide them into regions and analyze the signal characteristics at each stage of the corrosive process. Changes in signals were found depending on the stage, considering their amplitude, count, energy, rising time, and duration values [29,30]. Smanio et al. [24] developed a methodology to investigate individual acoustic sources in an SCC test, separating gas release, FeS (Iron Sulfide) formation, and hydrogen-induced cracking based on signal duration and energy. With this classification, it was possible to investigate the presence of each active source during the test time.

Calabrese et al. [31] adopted a new method for analyzing AE parameter data, such as principal component analysis and Kohonen neural networks with a self-organizing map (SOM). By integrating univariate and multivariate analysis into SOM, it was possible to identify the evolution and type of phenomenon occurring within a certain time interval, indicating crack initiation and propagation, critical material rupture, FeS formation, hydrogen gas evolution, and pitting initiation. In these studies, each phenomenon was identified, along with when it occurred and its position on the test specimen [31,32,33].

Some studies have also begun to attempt to identify not only the acoustic source but also to create methodologies to identify the severity of corrosion damage from AE signals. May et al. [34] propose a methodology for detecting corrosion in carbon steel structures at three different stages. Park et al. [35] sought to develop a real-time monitoring methodology for SCC, using only amplitude and energy to indicate the moment of crack initiation in a pipeline.

In this context, the development of real-time monitoring techniques capable of tracking the progression of hydrogen-induced damage stages as they occur is essential for advancing the understanding of material behavior. Such techniques enable researchers to gather critical information on the damage kinetics and the evolution of the cracking process, potentially offering additional valuable insights that traditional post-mortem analyses cannot capture. Then, this research explores the degradation and HIC of high-strength steel wires exposed to an H_2_S-saturated aqueous environment, following the NACE TM0284-2016 standard, using AE techniques. The wires, used in the tension armor of metal-based flexible pipes, were tested with two different exposure times and surface finishes to validate the degradation and damage mechanisms involved. To reinforce and confirm the AE findings, a comprehensive material characterization was conducted, including X-ray microtomography (micro-CT), optical microscopy (OM), and scanning electron microscopy (SEM). These methods enabled a detailed evaluation of the original microstructure and post-test identification of crack morphology and spatial distribution.

## 2. Materials and Methods

### 2.1. Material

**Figure 3:**
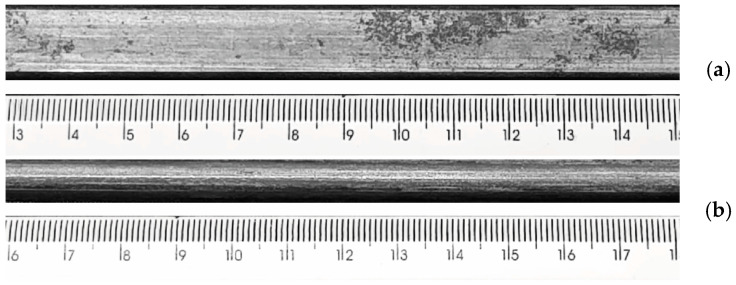
Photos of a section of the armor wire in as-received superficial condition. (**a**) top view and (**b**) side view. Scale in [mm].

Sections of armor wire were extracted from a metal-based flexible pipe and prepared for testing. The nominal dimensions of the section correspond to 6 mm × 25 mm × 300 mm. Figure 3 shows a section of the wire in as-received condition. The samples were tested as received and with surface preparation with 400-grit sandpaper.

The material’s hardness was evaluated using the Rockwell C scale in a MITUTOYO^®^ HR-430MS hardness tester equipped with a conical diamond indenter. The applied load was 150 kgf. The average hardness measured was 37 HRC.

The chemical composition of the wire, determined by arc/spark optical emission spectroscopy, is summarized in Table 1.

### 2.2. Corrosion Test

The corrosion tests were conducted based on the procedures outlined in NACE TM0284-2016 [10]. The tests employed solution A of the NACE TM0284 standard, consisting of 5.0 wt% NaCl and 0.05 wt% acetic acid dissolved in distilled (or deionized) water, prepared at room temperature. The solution was saturated with 99.9% pure H_2_S gas, resulting in an initial pH of 2.7. This solution fits within what are commonly known as sour environments [36].

**Figure 4:**
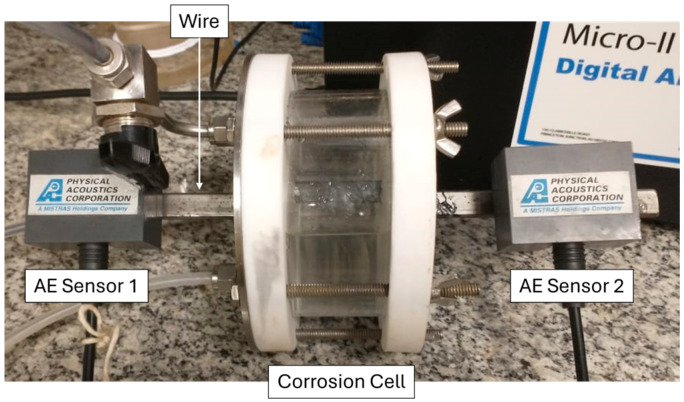
Photograph of the experimental setup showing the wire centered within the corrosion cell during the HIC test, with its ends connected to the AE sensors.

To enable real-time monitoring of cracking activity via AE, the test cell was configured to expose 60 mm in length of the central portion of the wire to the corrosive medium, while the ends remained accessible for AE sensors placement, as shown in Figure 4. The total duration of the first test was 96 h and the second, 24 h, both with a final pH of 3.5 and a dissolved H_2_S concentration of approximately 2400 ppm, as determined by potentiometric titration.

For this study, the results from two HIC tests are presented and discussed. The first test was conducted over 96 h on a wire in its as-received surface condition, referred to as Sample A. The second test involved a wire subjected to superficial treatment using 400-grit sandpaper, followed by exposure to the aggressive environment for 24 h; this specimen was designated as Sample B. The latter test was performed to support and confirm the findings and trends observed in Sample A.

### 2.3. Acoustic Emission

AE data were acquired using the Micro-II Express 8-channel system (Mistras Physical Acoustics), configured with two narrow-band PK15I sensors mounted at the ends of the sample outside the corrosion cell, as illustrated in Figure 4. The PK15I sensors operate in the 100–3000 kHz range, with a resonant frequency at 150 kHz. A bandpass filter from 100 to 400 kHz was applied to focus on the frequency range of interest. System acquisition settings were as follows: amplitude threshold of 38 dB, peak definition time (PDT) of 200 µs, hit definition time (HDT) of 800 µs, hit lockout time (HLT) of 1000 µs, maximum signal duration of 1000 µs, and a sampling rate of 2 MHz. These parameters were selected to optimize sensitivity to cracking-related emissions while minimizing noise and irrelevant events.

To enable spatial discrimination of acoustic activity during testing, two sensors were used to conduct 1D hit localization. This method allowed the identification of the approximate position of acoustic emission events along the specimen length.

### 2.4. Micrography and X-Ray Micro-Computed Tomography (Micro-CT)

After the HIC tests, the section of the sample exposed to the sour solution was cut and Micro-CT images were obtained to create 3D maps of the cracks within the sample.

The Micro-CT scan was performed using a V|tome|x M X-ray microfocus computed tomography system (Baker Hughes GE). Scan parameters were set at a voltage of 100 kV, current of 200 µA, acquisition time of 500 ms, 5 frames per projection, and a 1 mm thick Al filter placed at the X-ray exit window. A total of 1500 projections were acquired over a 360° rotation, and the voxel size resolution was 50.30 µm. Postprocessing and image analysis were conducted in the software Dragonfly 2025.

Additionally, the armor steel wire samples were analyzed using SEM and OM. SEM observations were conducted with a Vega 3 Tescan microscope and a ZEISS EVO MA 25 microscope, while OM analysis was performed using a ZEISS Imager.M1m microscope equipped with an AxioCam MRc 5 camera.

## 3. Results

### 3.1. AE Results

#### 3.1.1. Sample A (96 h)

**Figure 5:**
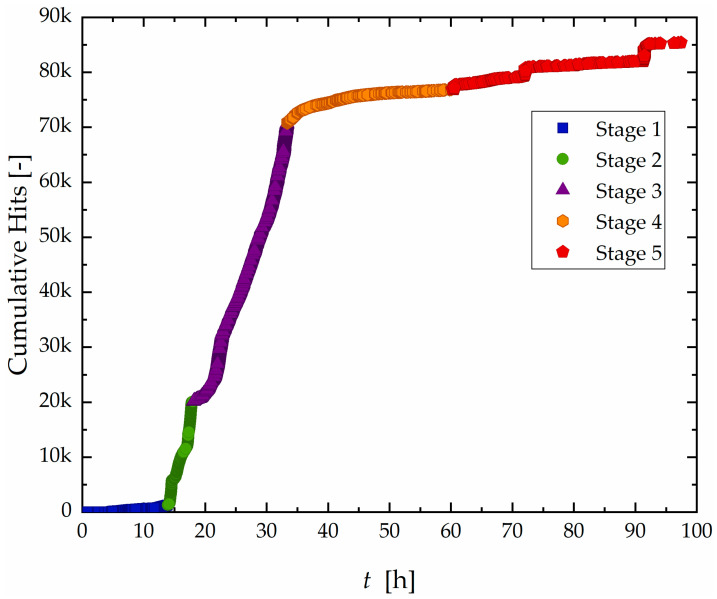
Curve of accumulated hit count as a function of time with division into five different stages for the Sample A.

The results of monitoring of AE activity during the test of sample A are shown in Figure 5, which presents the cumulative hit count over time.

Based on the signal accumulation rate, the test was divided into five distinct stages:

- Stage I (0–14 h): A period of low activity, with sparse AE hits and a gradual slope in the cumulative curve.
- Stage II (14–18 h): A sharp increase in AE activity, representing the highest hit rate observed during the test.
- Stage III (18–33 h): Although still active, the hit rate decreases compared to Stage II, indicating a transitional phase
- Stage IV (33–60 h): A progressive decline in hit count, with occasional localized bursts of activity.
- Stage V (60–96 h): Overall activity remains low, but distinct step-like increases in cumulative hits suggest the occurrence of isolated high-energy events.

This separation into stages reflects characteristic temporal changes in signal behavior, similar methodology employed in previous studies to classify corrosion progression based on AE data [23,27,29,31,37].

**Figure 6:**
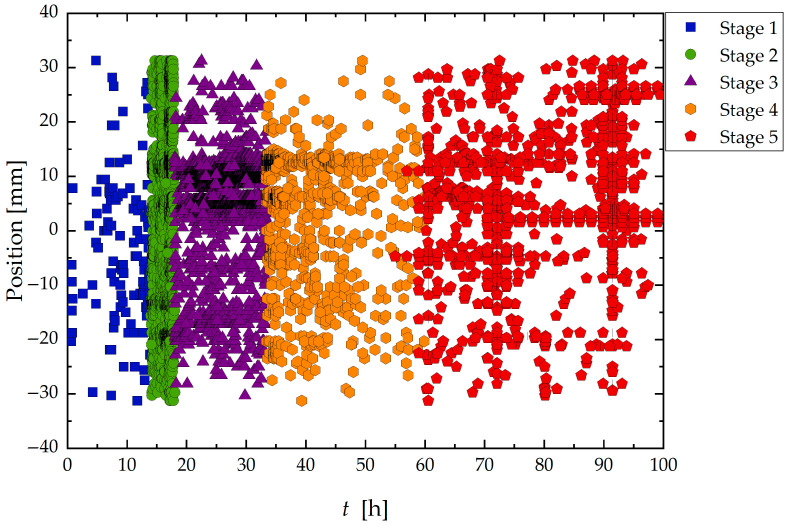
Localization of signals generated during the corrosion test according to time and classified into stages from 1 to 5.

Complementary to the temporal analysis, Figure 6 shows the spatial distribution of AE hits within the exposed region of the sample for each stage.

**Figure 7:**
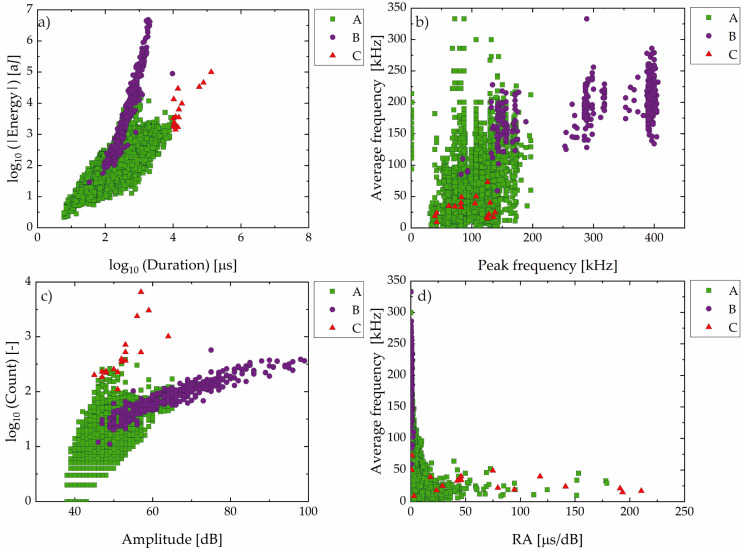
Classification of AE events for Sample A in Group A (green square), B (violet circle), and C (red triangle): (**a**) absolute energy vs. duration (log–log scale); (**b**) average frequency vs. peak frequency; (**c**) counts vs. amplitude (log–linear scale); (**d**) average frequency vs. RA (rise time/amplitude ratio) value.

Following the analysis of the temporal distribution and 1D localization of the events, the subsequent step consisted of evaluating and classifying the events regarding different the AE signal parameters. Based on the distributions of the signal parameters such as energy, duration, frequency, amplitude, count, and RA; the AE signals were classified into three groups, A, B, and C, as shown in Figure 7, using objective threshold criteria derived from time, frequency, and amplitude domains:

- Group C: Signals with duration greater than 10^4^ µs, typically associated with long-lasting and high-energy events.
- Group B: Signals with peak frequency above 250 kHz or amplitude greater than 65 dB, indicating high-intensity transient phenomena.
- Group A: Signals that do not meet the criteria for Groups B or C, comprising the remainder of the dataset.

These classification thresholds were chosen to reflect distinct signal behaviors observed during different stages of the test and are consistent with previous studies that correlate AE parameters with damage mechanisms in high-strength steels exposed to sour environments [16,24,37].

Group A (green square) signals, characterized by low to intermediate values of duration, energy, frequency, and low amplitude and rise time/amplitude ratio (RA), were the most numerous. In contrast, Group B (violet circle) consisted of signals with intermediate durations, but notably higher energy, amplitude, and frequency content, along with low RA values.

Group C (red triangle) includes signals with the highest values of duration, energy, and counts, but lower average and peak frequencies, and high RA values. These signals are less frequent and occur in bursts during the final stages of the test (Stage V).

**Figure 8:**
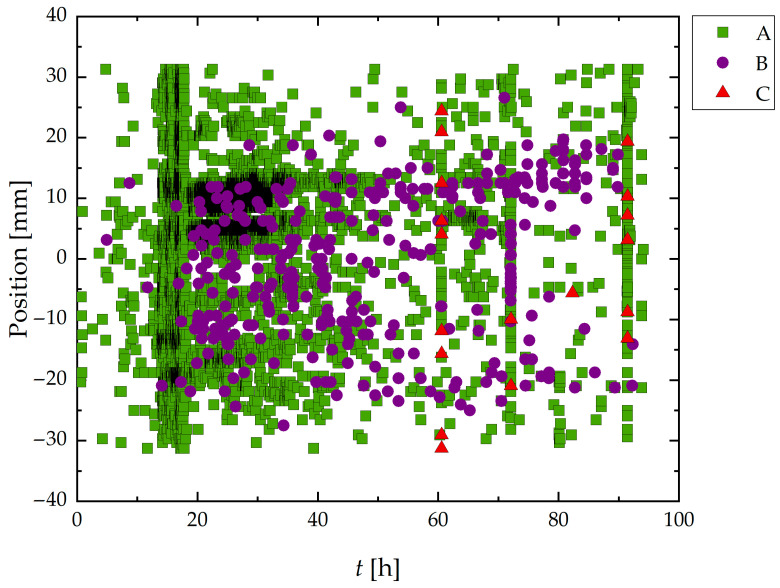
Spatiotemporal distribution of classified AE signals during HIC test of Sample A. The horizontal axis represents the elapsed time (h), and the vertical axis shows the position of each AE event along the wire (mm). Signals are classified into Group A (green triangles), Group B (violet squares), and Group C (red triangles), according to their waveform parameters. Zero position corresponds to the center of the exposed area of the wire to the sour environment.

Figure 8 presents the spatiotemporal distribution of AE hits during the HIC test of Sample A. Group A signals, which represent most recorded events, were broadly distributed in both time and space.

Analyzing together the results from Figure 7 and Figure 8, it was noted that Group B signals appeared more frequently between 15 and 70 h, with a tendency to cluster spatially around specific zones, particularly in the central portion of sample A. Also, Group C signals, though sparse, were clearly concentrated in narrow bands of time and space, primarily after 60 h. It is also important to note the vertical alignment of high-density events for Groups B and C, especially around 65 h and 85 h.

#### 3.1.2. Sample B (24 h)

**Figure 9:**
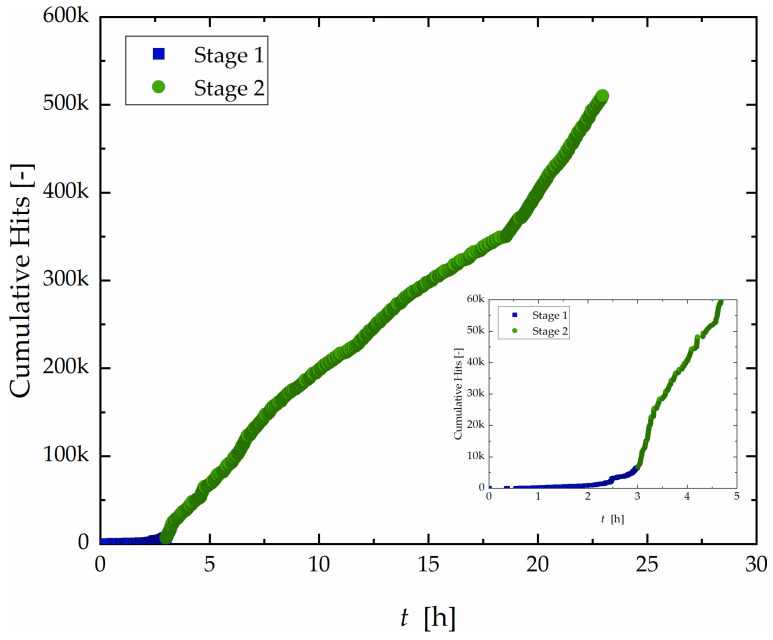
Curve of accumulated hit count as a function of time with division into two different stages for Sample B.

The cumulative hit count over time for the 24 h test is shown in Figure 9.

**Figure 10:**
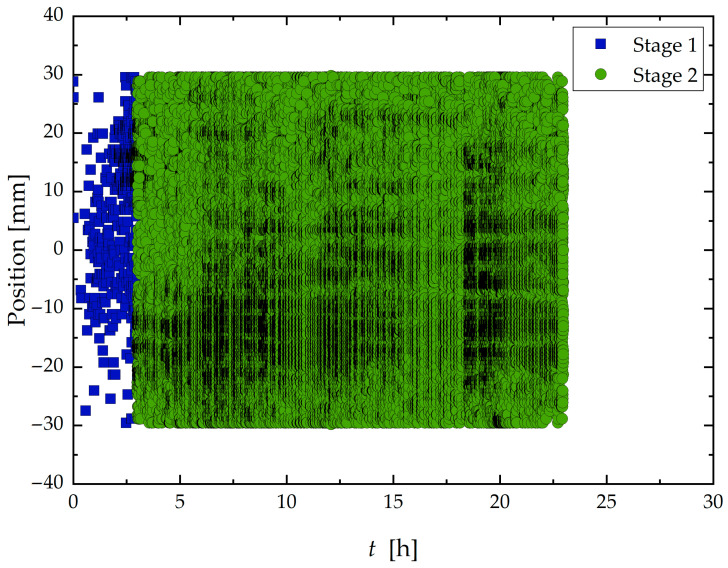
Localization of signals generated during the corrosion test according to time and divided into stages from 1 to 2.

Sample B (24 h) exhibited only the two initial discernible stages. The overall increase in acoustic activity compared to Sample A (96 h) at the initial stage is evident. The initial Stage I, typically defined as the incubation period, was significantly shortened to 3 h before transitioning into Stage 2, characterized by high activity. Moreover, as shown in Figure 10, these two stages exhibited spatial distributions like those observed in Sample A. Stage I was characterized by sparse and scattered hits across the surface, while Stage II displayed intense and widespread activity along the entire exposed area.

**Figure 11:**
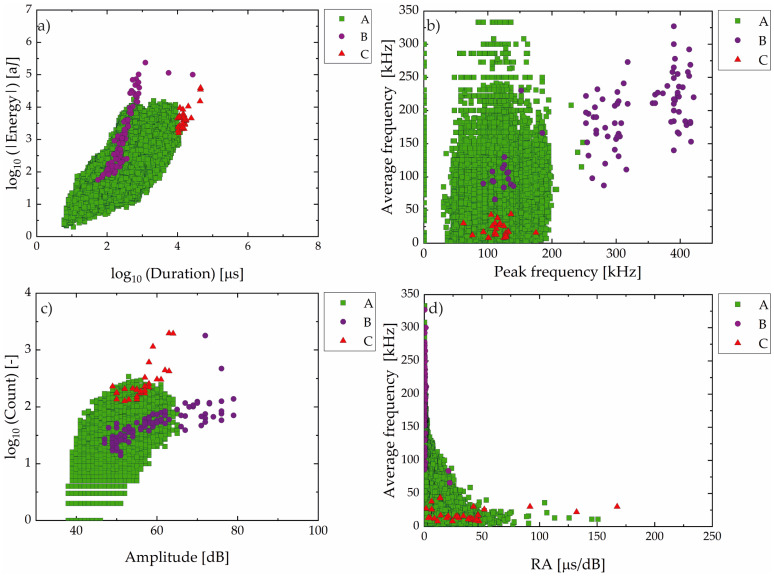
Classification of AE events for Sample B in Group A (green square), B (violet circle), and C (red triangle): (**a**) absolute energy vs. duration (log–log scale); (**b**) average frequency vs. peak frequency; (**c**) counts vs. amplitude (log–linear scale); (**d**) average frequency vs. RA (rise time/amplitude ratio) value.

Like the analysis of the AE parameter made in sample A, Figure 11 presents the classification of AE events from Sample B. A clear predominance of Group A signals was observed.

**Figure 12:**
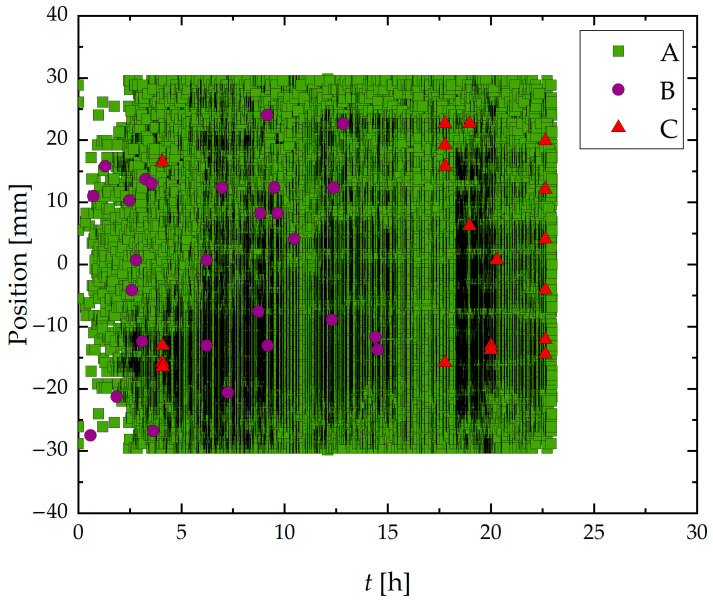
Spatiotemporal distribution of classified acoustic emission (AE) signals during the 24-h corrosion test on a ground steel wire specimen. The horizontal axis represents time (h), and the vertical axis shows the spatial position (mm) of AE events along the exposed segment of the wire. Data corresponds to Group A (green square), B (violet circle), and C (red triangle).

Figure 12 presents the spatiotemporal distribution of AE hits recorded during the HIC test of Sample B. These results revealed a marked increase in Group A signal density and a more homogeneous distribution along the length of the sample compared to Sample A, which retained its as-received surface condition.

In addition to Group A signals, a smaller number of Group B and Group C events were also recorded, albeit less frequently and concentrated mainly in the later hours of the test, particularly Group C signals.

### 3.2. Micro-CT Results

**Figure 13:**
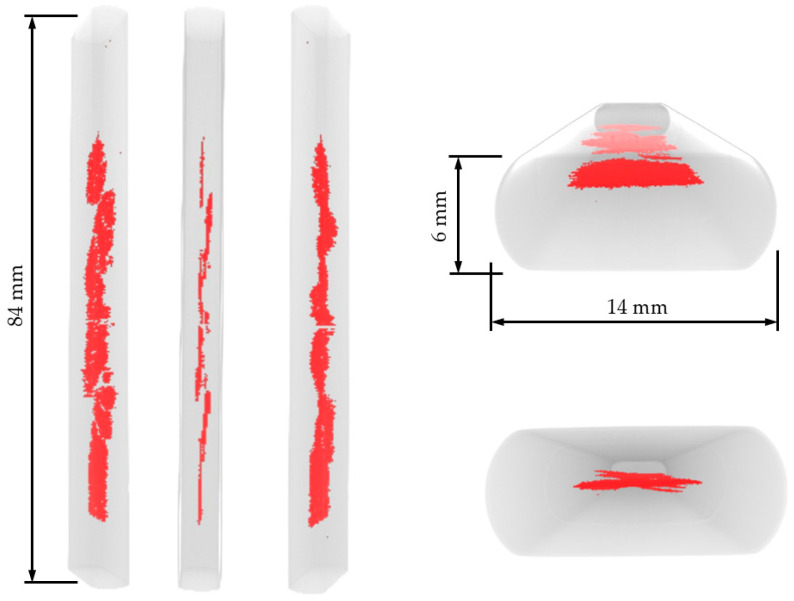
3D reconstruction views of Sample A after the test. Red highlighted region corresponds to internal cracks.

Figure 13 displays 3D reconstructions and selected tomographic slices obtained after exposing Sample A to the sour environment. Both the 3D reconstruction and the tomograms reveal significant cracking throughout the specimen. Despite some variation along the sample length, the cracks were predominantly concentrated at the central region, at mid-thickness, and presented approximately planar morphology.

**Figure 14:**
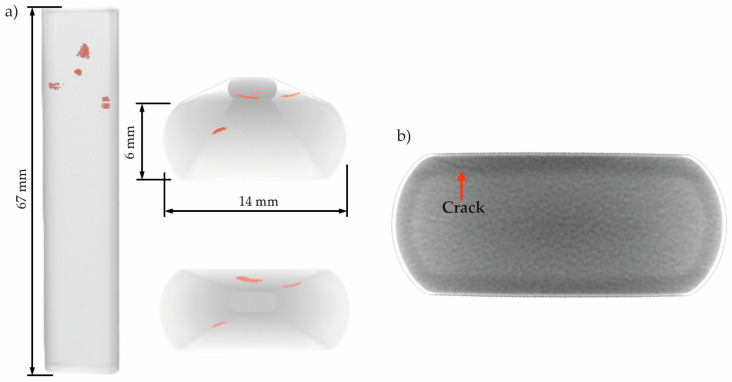
(**a**) 3D reconstruction views of Sample B after the test, and (**b**) a tomogram showing an HIC crack. Red highlighted region in (**a**) corresponds to internal cracks. The orange arrow in (**b**) highlights a crack.

Figure 14 presents the 3D reconstructions and a tomogram obtained for Sample B after exposure to the sour environment. 3D reconstructions in Figure 14a reveal only five small cracks located near the surface of the specimen. However, the tomogram in Figure 14b shows the presence of an additional small cracks, as an example of cracks distributed along the length of the sample that were not successfully segmented by the algorithm. Compared to Sample A, the cracks in Sample B were notably smaller and less prominent.

### 3.3. Microscopic Analyses

**Figure 15:**
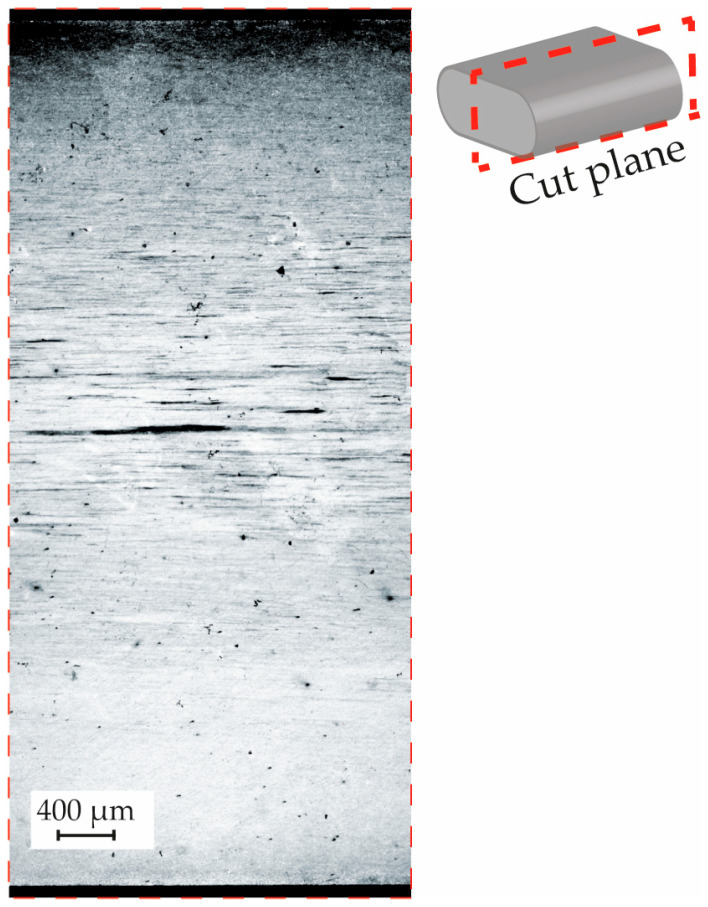
Metallography of the armor wire studied, machined from one of the ends of sample A. Etched with saturated picric acid.

To correlate the armor’s microstructure with the characteristics of the cracks observed after exposure to the sour environment, a metallographic examination was performed on the material. Figure 15 displays a micrograph taken on a plane perpendicular to the transverse section of the armor. A key observation was the presence of a segregation concentrated at the center of the wire.

**Figure 16:**
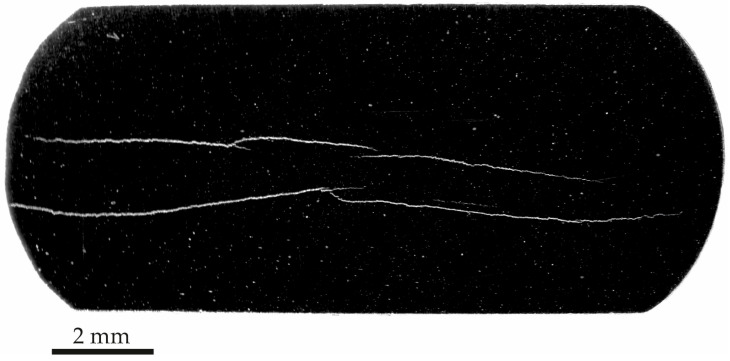
Photograph of the transversal section of Sample A after HIC test.

Following the HIC test, the samples were sectioned to allow inspection of the transverse plane of the armor wire. Figure 16 displays a representative section from Sample A. These results confirm the trend observed in the tomographic analysis, particularly regarding the nucleation and evolution of prominent cracking. The cracks were concentrated in the central region of the wire and extended horizontally across nearly the entire cross-section. It should be noted that the images were acquired at low magnification.

**Figure 17:**
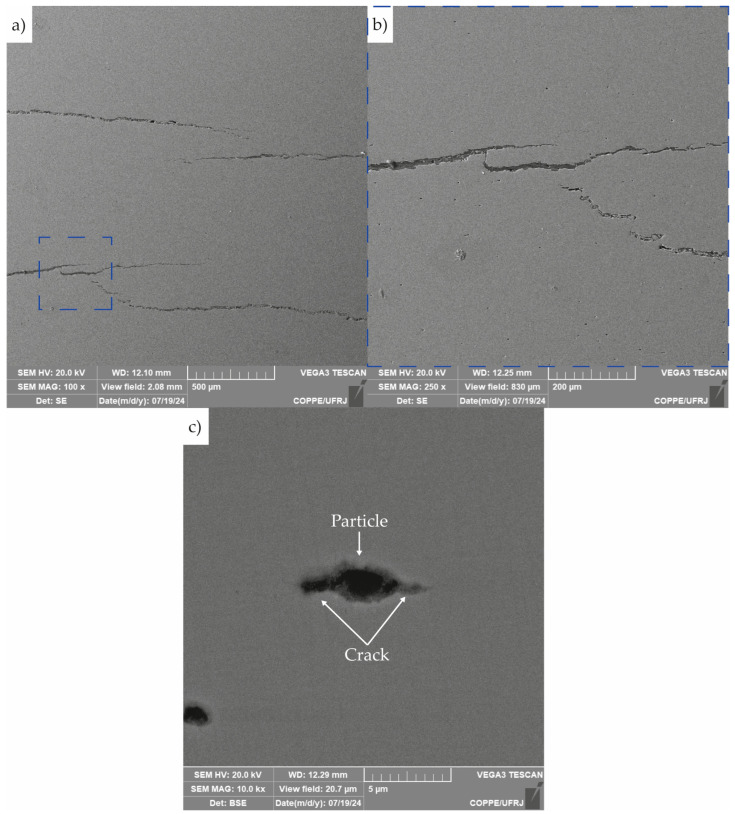
Scanning electron micrographs of crack in sample A: (**a**) crack at the central region of the sample, (**b**) zoom of the area highlighted by the blue dashed square in (**a**), and (**c**) the nucleation of a crack from a second-phase particle.

Micrographs of the cracks observed in the section shown in Figure 16 are presented in Figure 17. Figure 17a,b reveals stepwise cracking behavior, along with the presence of crack branching and directional changes. Figure 17c highlights the nucleation of a crack originating from a second-phase particle.

**Figure 18:**
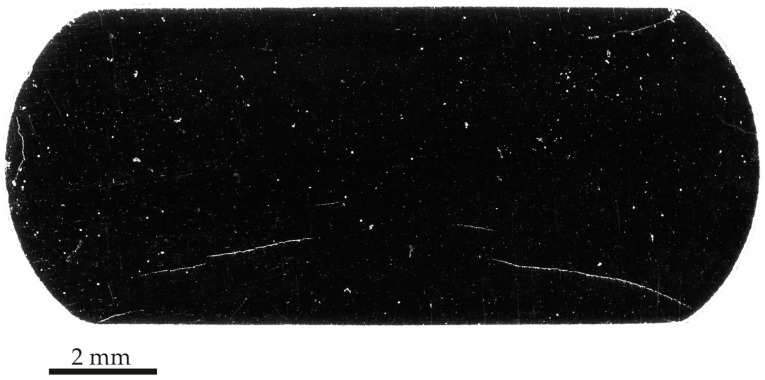
Photograph of the transversal section of sample B after HIC test.

On the other hand, Figure 18 shows the transversal section of Sample B after the HIC tests. In contrast to Sample A, Sample B exhibited less developed and smaller cracks, as well as some cracks near to the sample surfaces.

**Figure 19:**
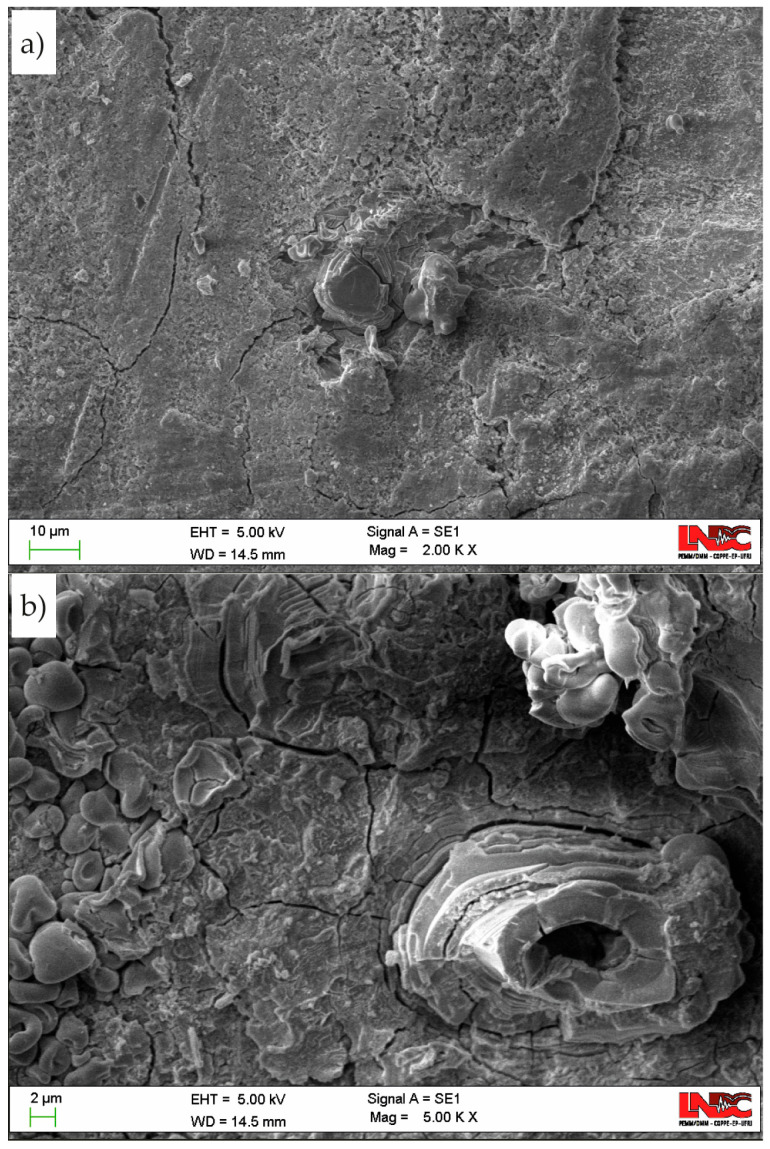
Scanning electron micrographs of the surface of Sample B after the HIC test obtained at a magnification of (**a**) 5000× and (**b**) 2000×.

After the HIC test, the surface of Sample B was examined to assess corrosion behavior. Figure 19 displays the corrosion products formed after 24 h of exposure to the sour solution. The image reveals a complex corrosion layer characterized by multiple morphologies and clear evidence of substantial surface cracking.

## 4. Discussion

### 4.1. Tomography and Microscopy Analyses

The results from both tomography and microscopy of Sample A revealed significant HIC in the studied armor wire after 96 h of exposure to the sour environment. In particular, the metallography in Figure 15 allows a clear correlation between microstructural segregation lines and the concentration of HIC cracks in the central region of the wire in Figure 16. This suggests that localized compositional variations may have played an essential role in crack nucleation and propagation under sour service conditions, in agreement with evidence in the literature [38,39,40], explaining the significant propagation of the cracks along the horizontal direction in Figure 16 [40]. However, other factors, such as those associated with the fabrication process, which may be linked to this observation, were not considered in the present analysis but could play an important role [41].

Figure 17c illustrates the nucleation of a hydrogen-induced crack from a second-phase particle. These particles are known to act as hydrogen trapping sites, where atomic hydrogen accumulates at the particle/steel matrix interface [41,42,43]. This localized accumulation facilitates hydrogen recombination, leading to internal pressure buildup. If hydrogen continues to accumulate, the rising pressure can initiate and propagate cracks. The microstructural evidence observed in Figure 17c strongly suggests that this HIC mechanism was activated in the studied armor wire.

The stepwise morphology of the crack observed in Figure 17a,b is characteristic of HIC. This pattern is typically attributed to crack coalescence during the HIC process [44]. During HIC, multiple small cracks may nucleate from different sites and subsequently merge. Additionally, crack tips can deflect and branch, contributing to the irregular, stepped appearance. These features are indicative of an advanced stage of HIC, reflecting significant material degradation and complex crack evolution mechanisms related to microstructural characteristics of the material.

To further investigate the origin of acoustic signals and gain deeper insight into the HIC process in the studied armor wire, as well as to confirm the trends observed in Sample A, the surface of Sample B was manually ground with 400-grit abrasive paper prior to testing. This surface preparation aimed to expose the metallic substrate and remove any passive films or oxides that could inhibit surface reactions. It is worth noting that oxide layers were present in the as-received condition, as shown in Figure 3.

After 24 h of exposure, tomographic and micrographic analyses of Sample B confirmed the presence of cracking, in agreement with the prediction made from the AE results of Sample A. However, as expected, HIC was less pronounced than in Sample A. The 3D reconstruction shown in Figure 14 highlights the challenges associated with segmenting the cracked regions in Sample B, which are primarily attributed to the limited crack development and the size of the sample scanned. The segmentation of brittle, plastically undeformed cracks is particularly challenging [45]. Figure 14b illustrates the lack of contrast between the bulk material and a crack; a limitation associated with the resolution employed and the extremely narrow gap between crack surfaces. Consequently, under the specific scanning conditions and voxel resolution used, these early-stage microcracks were more difficult to detect and accurately segment.

Visual surface examination of Sample B after the HIC test revealed a macroscopically uniform layer of corrosion products along the sample length. The corrosion process at the surface plays a critical role in HIC. In sour environments, the anodic dissolution of iron facilitates the reduction of hydrogen ions to atomic hydrogen, which can then be absorbed into the bulk material [44].

H_2_S serves as a hydrogen source in this process. While atomic hydrogen is inherently thermodynamically unstable, H_2_S inhibits its recombination into molecular hydrogen, thereby enhancing the absorption of atomic hydrogen into the steel matrix and promoting the HIC mechanism [44].

The SEM images in Figure 19 revealed a complex corrosion layer composed of diverse morphologies, indicating heterogeneous surface degradation. In sour environments, the corrosion process of iron-based alloys is generally understood to proceed as follows:

(1) $$Fe\;+\;H_{2}2(aq)\;\to \;{Fe}_{2}S\;+\;H_{2}$$

However, the sulfur-containing compounds formed during exposure are considerably more complex than those described by Reaction (1) [46,47]. While a detailed characterization of the corrosion layer is beyond the scope of this study, Figure 19 highlights the formation of these layers and provides clear evidence of their cracking.

As will be discussed in the following section, the surface treatment applied to Sample B resulted in increased corrosion activity compared to Sample A. This observation helps explain the contrasting presence of cracks near the surface of Sample B. Enhanced corrosion can lead to more substantial surface discontinuities, which may act as preferential nucleation sites for HIC [48].

### 4.2. AE Results

#### 4.2.1. Monitoring HIC in Sample A

Group A signals are typically associated with low-intensity surface phenomena, such as the formation and detachment of hydrogen bubbles and the precipitation or rupture of the corrosion layer on the metal surface. This interpretation is consistent with previous findings in corrosion environments containing H_2_S [21,23,49,50]. It should be noted that corrosion layer formation and its cracking process were evidenced by analyzing the surface of Sample B in Figure 19.

Additionally, in more advanced stages of HIC, signals from this Group A may also originate from localized plastic deformation at crack tips, particularly during early-stage microcrack development [16,51].

Regarding Group B signals, several studies [16,20,52,53,54,55] have correlated the characteristic signal of this group with the initiation and early propagation of microcracks, particularly under the influence of HIC. The presence of higher peak and average frequencies, as well as elevated hit counts, reinforces the interpretation that these are transient fracture events, likely occurring when hydrogen accumulates at microstructural discontinuities and facilitates crack nucleation. This micromechanics was evidenced by the micrograph in Figure 17c.

Previous works have linked Group C signals to unstable crack propagation, void coalescence, and ligament rupture, typical of the advanced phases of HIC [23,40]. As discussed in the previous section, this propagation process was evidenced in Sample A due to the stepwise morphology observed in Figure 17a,b.

Furthermore, a reduction in peak frequency during the propagation stage has been presented in Group C by various authors [56,57]. The trend of RA vs. average frequency observed in Figure 7d further supports the change in fracture phenomena. At early stages, signals exhibit low RA and high frequency, typical of crack opening. As the damage progresses, signals shift toward higher RA and lower frequency, which is indicative of propagation, often associated with crack coalescence and final failure. This behavior aligns with studies by Mukhopadhyay et al. [16], Kordatos et al. [58], and Calabrese et al. [59], and confirms that AE signal evolution can effectively capture the transition from crack initiation to unstable propagation under hydrogen-assisted degradation.

The trend observed in Group B regarding its special and time distribution (see Figure 7 and Figure 8) correlates well with crack-affected regions identified in the micro-CT 3D reconstruction, suggesting a strong association between Group B activity and early-stage crack initiation and propagation.

As mentioned above, Group C signals, though sparse, were clearly concentrated in narrow bands of time and space, primarily after 60 h. Their localized nature and delayed appearance are indicative of unstable crack growth and final-stage ligament rupture, aligning with previously described damage mechanisms in HIC [23,37,56,60,61], and the step-wise morphology observed in Figure 17a,b. Notably, the vertical alignment of high-density events for Groups B and C, especially between 65 h and 85 h, coincides with the stepwise increases observed in the cumulative hit curve in Figure 5, further supporting their relationship with accelerated crack evolution.

Results in Figure 8 indicated by the numerous events and spatial distribution of Group A that these signals reflect background processes such as hydrogen bubble formation, corrosion layer evolution, and possible low-intensity microstructural interactions.

The combination of temporal and spatial analysis in Figure 7 and Figure 8 highlights the effectiveness of AE signal classification in distinguishing between corrosion and HIC phenomena throughout the test.

#### 4.2.2. Monitoring HIC in Sample B

At this point, it is important to recall that Sample B was tested to verify the experimental trend observed in Sample A, which indicated the onset of HIC before 24 h of testing. This sample was superficially treated by grinding. As shown in Figure 11, the events were predominantly classified in Group A, which strongly supports the hypothesis that this category originates primarily from superficial electrochemical phenomena, such as hydrogen gas evolution and formation/rupture of corrosion layers, as evidenced in Figure 19. This interpretation is further reinforced by the shorter stage of low AE activity (only 3 h, much less than 14 h observed in Sample A in Stage 1), likely due to the direct exposure of the metallic surface after grinding.

The contrast observed in the distribution of Group A signals between Sample A and Sample B confirms the surface-related nature of these AE events, aligning with previous findings reported in the literature [21,23,37,49,50]. Thus, the marked heterogeneous activity observed along the length of sample A (see Figure 6 and Figure 8) was associated with the surface condition of this sample.

The distribution of Group B and Group C signals during the Sample B test suggests that, even under relatively short exposure times, localized microstructural degradation can initiate and trigger higher-intensity AE events, particularly when surface grinding increases susceptibility. This early damage hypothesis was confirmed by the observation of cracks in Figure 14, which are also consistent with the results from Sample A, indicating the onset of HIC before 24 h.

Figure 9 and Figure 12 also evidenced that due to the limited test duration (24 h), there was insufficient time for the transition into a third stage in Sample B, where activity becomes more localized. This is evidenced by the frequency and spatial distribution of hits, which remained diffuse and continuous rather than clear clustering into specific zones.

Considering the results from Samples A and B, it was demonstrated that the AE technique could monitor the HIC process throughout the test. This enabled the discrimination of the different stages of corrosion and damage evolution, as well as the one-dimensional localization of AE events within the sensor configuration employed. This capability is particularly relevant, as it provides important additional insights into the kinetics of the HIC process and the spatial development of damage, thereby contributing to a deeper understanding of hydrogen embrittlement phenomena.

### 4.3. Advantages of AE Monitoring to Evaluate HIC Susceptibility

Based on the analysis of AE results monitoring samples A (96 h) and B (24 h), exposed to a corrosive medium with 2400 ppm of dissolved H_2_S and pH 2.7, it was possible to record the time required for nucleation, growth, and propagation of cracks. Furthermore, it became evident that on an active, oxide-free surface, the cracking process begins much earlier. This information is highly relevant for predicting the behavior of materials under different exposure conditions. In general, wires in flexible pipes already have oxides on their surface; the results presented in this work show that in this scenario, even at high H_2_S concentrations and acidic pH, the onset of cracking is delayed compared to an oxide-free surface. Monitoring EA during the HIC testing enables researchers to analyze cracking kinetics and identify the specific variables influencing the process. The test protocol described herein is especially valuable to study the crack susceptibility on short-term exposures caused by failures or abrupt operational changes. With AE monitoring one can easily infer whether the H_2_S exposure was able to induce HIC or not.

Beyond the advantages offered by real-time monitoring in long-term tests presented in the introduction, this technique can also provide additional information in fixed-duration tests, such as NACE TM0284-2016 [10]. Although this standard does not specify acceptance or rejection criteria, AE monitoring can yield relevant information on corrosion and cracking processes, including event type, location along the specimen, and event intensity, which could also provide valuable insights about the material performance in the sour environment.

## 5. Concluding Remarks

This study demonstrated the application and effectiveness of the AE technique in monitoring HIC in high-strength steel wires used in armors of metal-based flexible pipes. Through the classification of AE signals based on their parameters, it was possible to associate distinct signal groups with specific physical mechanisms, including hydrogen bubble evolution, formation and rupture of the corrosion layer, and crack initiation and propagation.

The grouping of the test into activity-based stages, combined with spatial localization of AE events, enabled a detailed understanding of the progression of damage throughout the exposure period. The complementary 24 h test on a wire with ground surface (Sample B) further confirmed that Group A signals originate from superficial electrochemical phenomena (corrosion), given the high density of early signals and reduced initial low-activity stage.

Macroscopic metallographic analysis and micro-CT confirmed the presence of cracks in the wires, validating the signal classification methodology and reinforcing the capability of AE to detect and track internal degradation in real time. These results confirm that AE is even sensitive to the early stages of cracking and further evolution of HIC process.

Altogether, this work demonstrates that AE monitoring provides a disruptive alternative to traditional post-mortem corrosion tests, enabling the real-time detection of damage evolution and potentially allowing for test durations to be optimized, and providing valuable information about the mechanics HIC phenomena and its kinetics.

## Figures and Tables

**Table 1:** Chemical composition of the wire amor studied in wt%.

**C**	**Si**	**Mn**	**P**	**S**	**Cr**	**Ni**	**Mo**	**Al**	**Cu**
0.68	0.20	0.80	0.013	0.0028	0.052	0.032	<0.002	0.003	0.008
**Co**	**Ti**	**Nb**	**V**	**W**	**Pb**	**B**	**Sn**	**Zn**	**Fe**
0.0024	<0.001	<0.003	0.003	<0.010	<0.003	<0.0005	0.001	<0.002	Bal.

## Data Availability

The original contributions presented in this study are included in the article. Further inquiries can be directed to the corresponding author.

## Abbreviations

The following abbreviations are used in this manuscript:

AE	acoustic Emission
HDT	hit definition time
HE	hydrogen embrittlement
HIC	hydrogen-induced cracking
HRC	hardness Rockwell C scale
micro-CT	computed X-ray microtomography
OM	optical microscopy
RA	rise time/amplitude ratio
SCC	stress corrosion cracking
SEM	scanning electron microscopy
SOM	self-organizing map
